# ALPS-like Disorder Linked with STAT3 Mutation: *De novo* Variant with Bicytopenia and Literature Review

**DOI:** 10.2174/0118715303470784260605093400

**Published:** 2026-06-22

**Authors:** Aylar Mohammadi, Zahra Hamidi Esfahani, Hassan Abolhassani, Samin Sharafian, Sabahat Haghi, Nima Rezaei, Reza Yazdani, Marzieh Tavakol

**Affiliations:** 1 Research Center for Immunodeficiencies, Pediatrics Center of Excellence, Children's Medical Center, Tehran University of Medical Sciences, Tehran, Iran;; 2 Division of Clinical Immunology, Department of Biosciences and Nutrition, Karolinska Institutet, Karolinska University Hospital, Huddinge, Stockholm, Sweden;; 3 Department of Allergy and Clinical Immunology, Mofid Children's Hospital, Shahid Beheshti University of Medical Sciences, Tehran, Iran;; 4 Department of Pediatrics, Imam Ali Hospital, Alborz University of Medical Sciences, Karaj, Iran;; 5 Primary Immunodeficiency Diseases Network (PIDNet), Universal Scientific Education and Research Network (USERN), Tehran, Iran;; 6 Non- Communicable Diseases Research Center, Alborz University of Medical Sciences, Karaj, Iran

**Keywords:** Inborn errors of immunity, primary immunodeficiency, bicytopenia, ALPS, autoimmunity, STAT3, gain-of-function

## Abstract

**Introduction:**

Autoimmune lymphoproliferative syndrome-like (ALPS-like) disorders are inherited conditions caused by non-FAS pathway mutations that clinically mimic ALPS, presenting with lymphoproliferation, autoimmunity, and cytopenias. This study describes a patient with STAT3-related ALPS-like disease that was initially misdiagnosed as ALPS and compares his clinical, immunological, and molecular features with those of previously reported cases to highlight diagnostic distinctions from classical ALPS.

**Methods:**

Clinical data were obtained from direct examination and medical records. Whole-Exome Sequencing (WES) identified the causative mutation. A literature review using PubMed, Web of Science, and Scopus retrieved previously reported ALPS-like cases with STAT3 mutations for comparative analysis of clinical, immunological, and molecular findings.

**Results:**

We report a 10-year-old boy with bicytopenia (thrombocytopenia and neutropenia), autoimmune thrombocytopenic purpura, refractory lymphadenopathy, splenomegaly, and recurrent infections, initially misdiagnosed as ALPS. Elevated double-negative T cells and vitamin B12 levels were detected. WES revealed a heterozygous *de novo* STAT3 mutation (p.L666V). A reduced frequency of Treg cells was observed in our case. The patient responded well to JAK inhibitor therapy. Review of reported STAT3-mutant ALPS-like cases (66 cases) showed that immune thrombocytopenia was the most common cytopenia, often accompanied by autoimmune hemolytic anemia, variable hypogammaglobulinemia, reduced Treg cells, and increased double-negative T cells.

**Discussion:**

STAT3 gain-of-function–associated ALPS-like disease can closely mimic classical ALPS due to overlapping clinical and immunological features, including elevated double-negative T cells, which may lead to diagnostic challenges.

**Conclusion:**

This case highlights that STAT3 GOF ALPS-like disease can closely mimic classical ALPS, and that integrating genetic analysis with immunological assessment is essential for accurate diagnosis and guiding targeted therapy.

## 1 INTRODUCTION

Autoimmune lymphoproliferative syndrome (ALPS) is a rare inherited disorder caused by mutations in genes of the FAS-mediated apoptosis pathway, including FAS, FAS ligand (FASL), Caspase 8 (CASP8), and Caspase 10 (CASP10). These mutations, which may be germline, somatic, or both, dysregulate the FAS apoptotic pathway, which plays a crucial role in lymphocyte homeostasis, thereby leading to chronic nonmalignant lymphadenopathy, splenomegaly, multi-lineage cytopenias, and an increased risk of lymphoma [1]. These mutations may occur in germline or somatic cells, and both types can dysregulate the FAS apoptotic pathway. This pathway plays a crucial role in lymphocyte homeostasis; its disruption results in chronic non-malignant lymphadenopathy, splenomegaly, multilineage cytopenias, and an increased risk of lymphoma. ALPS-like diseases are a group of genetic disorders that mimic the ALPS phenotype and are characterized by mutations outside the FAS pathway. They are associated with mutations in non-FAS-mediated apoptosis pathways [2, 3]. The most common manifestations of ALPS-like diseases are lymphoproliferation and autoimmune cytopenias. Recurrent infections, including lower and upper respiratory tract infections (viral and bacterial), are more frequent in ALPS-like patients than in ALPS patients [2, 4].

The signal transducer and activator of transcription 3 (STAT3) is a member of the STAT protein family that, in response to extracellular cytokines, interferons (IFNs), or growth hormones, regulates gene expression and controls apoptosis, proliferation, and cell growth. Germline STAT3 Gain-Of-Function (GOF) mutations underlie an ALPS phenotype and lead to recurrent infections, multi-organ autoimmunity, hypogammaglobulinemia, and lymphoproliferation [5]. STAT3 plays a crucial role in T cell development, and dysregulation of STAT3 impairs Treg-mediated immune tolerance, leading to autoimmunity in patients with ALPS-like disorders [6]. Given that STAT3 transmits cytokine signals from cell-surface receptors to the nucleus *via* Janus kinases (JAKs), JAK inhibitors are effective in treating STAT3 GOF patients with severe autoimmunity and immune dysregulation [7]. Elevations of double-negative T cell (DNT) (>1.5% of total lymphocytes or >2.5% of CD3^+^ T cells) and vitamin B12 (> 1500 ng/L) are useful serum biomarkers in the diagnosis of both ALPS and ALPS-like diseases [8, 9]. However, there are no definitive clinical or laboratory guidelines to differentiate ALPS from ALPS-like; therefore, genetic assessment is necessary [10].

We herein report the first case of a STAT3 GOF patient with lymphoproliferation and autoimmunity from the National Iranian Inborn Errors of Immunity (IEI) Registry [11], a case initially misdiagnosed as ALPS. Clearly distinguishing between ALPS and ALPS-like diseases is challenging but crucial for accurate diagnosis and appropriate therapy.

## 2 MATERIALS AND METHODS

### 2.1 Data Collection

Patient information, including demographic data, medical history, physical examination, and laboratory findings, was collected through direct interviews and review of the patient's medical documents after obtaining written informed consent from the patient and his parents. The current study was approved by the Ethics Committee of the Alborz University of Medical Sciences (IR.ABZUMS.REC.1404.144). Demographic data included age, gender, age at onset, age of diagnosis, and family history. The recorded laboratory data included complete blood counts, lymphocyte subsets, TCR αβ+ DNT (percentage and count), immunoglobulin levels, Lymphocyte Transformation Test (LTT), infection panels, thyroid markers, liver function tests, and vitamin B12 measurement.

### 2.2 DNA Extraction and Whole-Exome Sequencing

Genetic evaluation was performed on EDTA blood samples collected from the patient, the patient's parents, and a control. DNA from the proband was extracted, and whole-exome sequencing was performed on the Illumina NovaSeq as described previously [12, 13]. Variants were called, filtered by quality metrics, annotated, and analyzed for rarity and pathogenicity using population databases and predictive tools based on American College of Medical Genetics and Genomics (ACMG) criteria [14]. IEI genes were prioritized based on the updated IUIS (International Union of Immunological Societies) classification [15]. Sanger sequencing validation was conducted to confirm the candidate gene (STAT3), and segregation analysis was performed using the following primers: Forward: TCTGGCCGACAATACTTTCC and Reverse: TCCGTGGAACCATACACAAA.

### 2.3 Flow Cytometry

To enumerate Treg cells, peripheral blood mononuclear cells (PBMCs) were isolated from whole blood by using Ficoll Lymphodex (Innotrain, Germany) gradient centrifugation according to the manufacturer’s instructions. For extracellular staining, PBMCs were stained for 20 min at 2–8 °C in the dark with the monoclonal antibodies against CD4, CD127, and CD25 at optimal concentrations. By the aim of intracellular staining of Foxp3, after the surface molecule staining, cells were fixed and permeabilized by FOXP3/Permeabilization buffer (eBioscience, US), and cell frequencies were determined using a BD FACSCalibur flow cytometer (BD Biosciences). Lymphocytes were gated based on forward and side-scatter profiles, and Treg cells were identified by the CD4+, CD127low/-, CD25high, FoxP3+ phenotype. Flow cytometry analysis was performed using FlowJo software (version 10.10).

### 2.4 Literature Review

The literature search for reported ALPS-like patients with STAT3 GOF mutations was performed using PubMed, Web of Science, and Scopus. The following keywords were used: “Autoimmune lymphoproliferative-like syndrome” and “ALPS-like syndrome”, in combination with “STAT3”, and “The signal transducer and activator of transcription 3.” Based on the title and abstract, irrelevant studies were excluded. Cases without ALPS-like assignment were also included if patients presented lymphoproliferation (defined as lymphadenopathy, splenomegaly, hepatomegaly, or all three together) along with at least one form of autoimmune cytopenia, such as autoimmune hemolytic anemia (AIHA), Immune Thrombocytopenic Purpura (ITP), or Autoimmune Neutropenia (AN) [2].

## 3 RESULTS

### 3.1 Case Presentation

A 10-year-old Iranian male with a history of prolonged thrombocytopenia associated with neutropenia was referred to the immunology clinic for further diagnostic evaluation. He was the second child of non-consanguineous parents, with normal gestational age and up-to-date vaccination status. The patient had a history of recurrent aphthous stomatitis with no candidiasis, and also a history of recurrent gingivitis between 10 months and 4 years of age. He complained of dry skin from the early months of infancy, which had been clinically diagnosed as psoriasis without any observable skin lesions. There was a history of recurrent epistaxis, frequent common colds with prolonged nasal discharge, and a productive cough, all of which generally received outpatient treatment. He experienced some mild abdominal pains and a poor appetite since childhood, with no history of significant gastrointestinal problems or growth failure. The family history of the patient revealed an older brother with a history of lymphadenopathy that did not require hospitalization, and a paternal uncle who died at the age of two with an unknown reason.

At the age of 4 years, he was admitted to the hospital for the first time due to a two-week history of splenomegaly and unilateral lymphadenopathy. He underwent a needle biopsy of lymph nodes, but the pathology result was insignificant, and he was clinically diagnosed with EBV+ infectious mononucleosis. He had an episode of epididymo-orchitis at the age of 5, and a history of a scrotal cyst, which was surgically removed during infancy, and no pathology was available. The patient experienced thrombocytopenia at the age of 9, which was diagnosed as ITP and treated with intravenous immunoglobulin (IVIG). However, thrombocytopenia recurred several times. Treatment with prednisolone and IVIG was continued based on a possible diagnosis of chronic ITP. Later, he experienced 2-3 episodes of dental abscess between the ages of 9 and 10, and neutropenia was noticed. Bone marrow examination revealed decreased maturation in all three lineages, without increased blasts.

At the age of 10, he was referred to our immunology clinic due to the refractoriness to treatment and inability to discontinue steroids or IVIG. Laboratory tests indicated elevated levels of serum immunoglobulins (IgG, IgA, IgM, and IgE) and vitamin B12. Flow cytometry showed increased DNT in peripheral blood. In addition, LTT demonstrated reduced proliferative responses to Bacillus Calmette–Guérin (BCG), Diphtheria Toxoid (DT), and *Candida* antigens (Table **1**). Given the high suspicion of primary immunodeficiency, a genetic test was ordered and, as the patient’s clinical and laboratory data were consistent with ALPS, sirolimus was initiated while awaiting the definitive diagnosis.

The frequency of cytopenia dramatically decreased after starting sirolimus. However, the patient experienced transient cervical lymphadenopathy and neutropenia with or without thrombocytopenia occasionally, which was associated with low-grade fever and coryza symptoms compatible with viral upper respiratory tract infections. One of these episodes presented with high-grade, prolonged fever, anterior cervical and submandibular lymph node enlargement, in addition to splenomegaly, which resulted in hospital admission. Excision biopsy of the lymph node was requested. During the operation, the surgeon identified a submandibular mass that appeared more consistent with a salivary gland, although it was indistinguishable from lymph nodes. A biopsy showed severe lymphoid infiltration with large reactive germinal centers in the salivary gland tissue, favoring a benign reactive process. Given the persistence of lymphadenopathy despite the administration of sirolimus, the diagnosis of ALPS was reconsidered, and the patient’s genetic testing was followed to identify potential disease-causing mutations. Whole-exome sequencing was conducted on DNA extracted from PBMCs and ruled out all known ALPS genes within the FAS pathway and also all other known IEI genes. However, we identified a heterozygous *de novo* mutation in the SH2 domain of STAT3 (ENST00000264657.5: c.1996C>G, ENSP00000264657.4:p. Leu666Val, minor allele frequency of 0, Combined Annotation Dependent Depletion score: 26.2 > mutation significant cut-off 0.003, Fig. **1A**). This variant was most likely a germline mutation, with a variant allele frequency of 67 alternative reads out of 113 total reads at this locus (VAF: 59.2%). Sanger sequencing of both parents did not detect the variant, thereby confirming its *de novo* origin Fig. **1A**). Of note, IgE levels were slightly elevated but not at the level of Hyper-IgE syndrome. Given that patients with ALPS-like disease often exhibit defects in regulatory T (Treg) cells [8], flow cytometry analysis was performed to enumerate Treg cells. The results showed a reduced frequency of Treg cells (1.16%) in the patient's blood compared to the sex- and age-matched control (17.4%) (Fig. **1B**), further supporting the diagnosis of an ALPS-like disorder in this case. By confirming the diagnosis of ALPS-like disorder due to STAT3-GOF in this patient at the age of 11, treatment was initiated with a JAK inhibitor, tofacitinib 5mg/BD for 2 years and then 5mg daily, which completely controlled the symptoms of the disease. The patient continues to take tofacitinib as long-term maintenance therapy. He is now 14 years old, alive, and living a normal life, and has no longer experienced cytopenia or lymphadenopathy after starting tofacitinib. Serial complete blood count evaluations showed normalization of hematologic parameters, while post-treatment immunologic analyses were not repeated due to financial constraints.

### 3.2 Literature Search Results

The literature search revealed 66 patients with STAT3-related ALPS-like phenotype (53% male). The median (IQR) ages of symptom onset, diagnosis, and current age were 3 (1-7) years, 15 (10-25) years, and 18 (13-26) years, respectively. Review of the literature showed that seven patients (10.7%) had died. The global distribution of the reported ALPS-like patients with STAT3 GOF mutations is illustrated in Fig. (**2**). All patient demographic data are presented on a case-by-case basis in Table **2**. As shown in Table **2**, all patients experienced lymphadenopathy and splenomegaly. The frequencies of failure to thrive (FTT), skin diseases, viral infection, and bacterial infection in patients were 53% (35 cases), 42% (28 cases), 53% (35 cases), and 59.1% (39 cases), respectively. Among the types of cytopenias, ITP (81.85%) was the most common in the total patient cohort. The clinical presentation in ALPS-like patients with STAT3 GOF mutations is detailed in Table **3**.

Based on our literature review, all patients had a heterozygous missense mutation distributed across different STAT3 domains, including the DNA-binding and SH2 domains (SH2 domain mutations accounted for 4.5%). Considering laboratory findings, reduced Treg cells were documented in 14 out of 24 patients (58.3%). Among 47 patients with available DNT data, 40 (85.1%) patients showed an increase in DNT (≥ 1.5% of total lymphocytes or 2.5% of CD3+ lymphocytes), while 7 (14.8%) cases had a normal DNT number (Usually < 1% of total lymphocytes) (Table **4**). Most patients exhibited a hypogammaglobinemia phenotype (66.0%), while 2% had a Class Switch Recombination (CSR) defect, and 27% had normal immunoglobulin levels (Supplementary Figure **S1**). Regarding treatment, our literature search revealed that 21.2% (14 cases) of patients received JAK inhibitor therapy, 83.3% received immunoglobulin therapy, and only 5 patients required Hematopoietic Stem Cell Transplantation (HSCT). Among the 14 patients who received JAK inhibitors and the 4 patients who underwent HSCT, 3 and 2 patients died, respectively. Detailed information regarding treatment responses was not available in the literature (Table **5**).

## 4 DISCUSSION

Here, we described the first STAT3 GOF ALPS-like case from the national Iranian registry with recurrent lymphadenopathy and periodic episodes of bicytopenias (neutropenia and thrombocytopenia). The laboratory findings showed elevated DNT cells, low Treg, and increased IgG levels. Although elevated DNT cells are a hallmark of classical ALPS, they are not disease-specific and may also be observed in STAT3 GOF ALPS-like; therefore, their diagnostic significance should be interpreted in the context of additional immunological features and confirmed by genetic testing. While the patient had previously received IVIG and sirolimus, his clinical symptoms were completely controlled after initiation of JAK inhibitor therapy.

Patients with STAT3 GOF share common clinical features with ALPS patients, including lymphoproliferation and autoimmune cytopenias, and are categorized as having an ALPS-like phenotype [5]. Based on the latest European Society for Immunodeficiencies (ESID) criteria, ALPS is diagnosed when a patient has chronic (> 6 months), non-malignant, noninfectious lymphadenopathy and/or splenomegaly, along with elevated DNT cells, interleukin-10, vitamin B12, and soluble FasL (sFasL) (https://esid.org/Working-Parties/Registry-Working-Party/Diagnosis-criteria). Previous studies have revealed that splenomegaly (92%) and lymphadenopathy (86.8%) are the most common clinical findings in ALPS [3]. The conducted literature review showed that STAT3 GOF patients selected from ALPS-like cohorts consistently exhibit splenomegaly and lymphadenopathy; however, data on hepatomegaly were limited [3]. Similarly, our case also presented splenomegaly and lymphadenopathy [3]. These findings support the notion that splenomegaly and lymphadenopathy are common clinical features shared by both ALPS and ALPS-like disorders, including STAT3 GOF. Despite ALPS, recurrent infections are the most common clinical manifestation in ALPS-like patients [2-4]. Most ALPS-like patients with Treg defects experience viral and bacterial respiratory tract infections [2]. Our findings revealed that 53% and 60% of patients experienced viral and bacterial infections, respectively. This highlights the need to pay attention to infectious susceptibility, especially in the respiratory tract, as a prominent feature of patients with STAT3 GOF ALPS-like, particularly in patients with Treg defects [16].

Autoimmune cytopenia results from autoantibody-mediated destruction of blood cells and is aggravated by splenic sequestration [17]. Previous studies reported the occurrence of cytopenias in various subtypes of patients with ALPS-like disorders [2]. AIHA is the most frequent autoimmune cytopenia in ALPS, whereas ITP is most common in ALPS-like disorders [3]. Our review of patients with STAT3 GOF similarly showed that ITP was the most predominant type of cytopenia, with 24 patients experiencing bicytopenia, most commonly involving ITP and AIHA [4, 5, 7, 18-23]. Our case presented with bicytopenia (ITP and AIN), a pattern observed in 44% of previously reported cases with STAT3 GOF [4, 5, 7, 18-22, 24]. These findings underscore that patients with STAT3 GOF ALPS-like present a wide spectrum of cytopenias, with a predominance of ITP.

Germline STAT3 GOF mutations occur across all protein domains and affect multiple signaling steps, influencing clinical variability. Our systematic review reported ALPS-like patients with STAT3 mutations, correlating molecular effects with patient phenotypes [3]. It has been suggested that GOF mutations can be grouped into categories based on baseline DNA-binding or nuclear shuttling activity, with prolonged SH2 domain phosphorylation observed in our current proband [25]. These findings suggest that mutation-specific molecular changes affect disease severity and clinical features, though further studies are needed to validate these mechanistic patterns in a larger patient cohort. Somatic STAT3 mutations in T-cell large granular lymphocytic leukemia (T-LGLL) are recurrent and functionally activating, supporting a key role of STAT3 signaling in cytotoxic T-cell–mediated immune dysregulation [26].

Currently, no clinical or laboratory guidelines exist for the diagnosis of patients with ALPS-like disorders; therefore, enumeration of DNT and Treg cells should be performed in any suspected ALPS patients [27]. Although an increased number of DNT cells is a diagnostic hallmark of ALPS, almost half of patients with ALPS-like disorders exhibit elevated DNT cells [28], underscoring that elevated DNT cells cannot be considered diagnostic of ALPS-like disorders. Among patients with STAT3 GOF whose DNT cell counts were reported, a substantial proportion (81.2%) showed elevated DNT cell counts [4, 5, 7, 18-23]. Additionally, our review showed that 14 out of 24 patients (58.3%) with available data indicated reduced Treg cell levels in STAT3 GOF-associated ALPS-like disorders [4, 5, 7, 18, 20, 23, 29]. In contrast, Treg cell numbers in classical ALPS are usually normal, although functional alterations have been reported. These findings highlight that although elevated DNT cells and low Treg cells are common in patients with STAT3 GOF, they are not enough for diagnosis on their own. Instead, they should be considered supportive indicators, reinforcing the importance of integrating genetic testing with immunophenotyping in the diagnostic workup.

There is no distinct treatment strategy for ALPS-like disorders, and the approach is similar to what is generally utilized for patients with a probable diagnosis of ALPS [9]. The first-line treatments of ALPS and ALPS-like involve improving complications and clinical manifestations as a palliative therapy. Initial therapy for autoimmune cytopenia is high-dose corticosteroids with/or without IVIG. To treat refractory cytopenias, sirolimus, an mTOR (mammalian target of rapamycin) inhibitor, has been used as a second-line treatment, which acts by inducing apoptosis of DNTs and promoting the development of regulatory T cells [30]. It is also effective in non-ALPS patients with autoimmune cytopenias [31]. In our patient, clinical manifestations were well controlled after initiation of a JAK inhibitor, whereas sirolimus was ineffective. Most of the reported patients with STAT3 GOF received IVIG as a first line. Among the 14 patients who received JAK inhibitors and the 4 patients who received HSCT, only 3 and 2 patients died, respectively [4, 7, 29]. These observations suggest that although conventional therapies such as corticosteroids, IVIG, and sirolimus are effective, low efficacy in some patients underscores the importance of targeted therapies, such as JAK inhibitors, for patients with STAT3 GOF.

This study had some limitations. First, the pathogenicity of the identified STAT3 variant in the proband is inferred solely from in silico predictions and the clinical phenotype; future functional assays are needed to confirm its GOF effect *in vitro*. In addition, the primary findings of this study are derived from a single patient, which limits the generalizability of the clinical, immunologic, and genetic observations and precludes definitive conclusions regarding broader genotype–phenotype correlations in STAT3 GOF ALPS-like disease. Although we collected all reported patients with STAT3 GOF based on available electronic data, some relevant cases, especially non-English sources, may not have been captured. Additionally, immunologic and laboratory parameters were not uniformly reported across the included studies, and immunologic data were missing in several reports; therefore, analyses were performed based on available data, which may limit definitive conclusions regarding immunologic patterns in patients with STAT3 GOF ALPS-like disease. Furthermore, variable treatment protocols and follow-up across studies limit our ability to draw a definitive conclusion about treatment efficacy and long-term outcomes.

## CONCLUSION

ALPS and STAT3 GOF ALPS-like disorders share overlapping clinical features, including lymphadenopathy, splenomegaly, and autoimmune cytopenias, which may complicate differential diagnosis based on clinical and immunological findings alone. However, distinct genetic and immunophenotypic characteristics can help differentiate these entities. Our experience highlights that genetic testing is essential not only to establish an accurate diagnosis in patients presenting with an ALPS phenotype but also to guide appropriate therapeutic strategies. In this case, confirming STAT3 GOF ALPS-like disease enabled targeted treatment with a JAK inhibitor, resulting in complete control of clinical symptoms. Therefore, integrating comprehensive immunological evaluation with molecular analysis is critical for precise diagnosis and personalized treatment. Further studies are warranted to refine the clinical and biological distinction between these conditions.

## AUTHORS’ CONTRIBUTIONS

The authors confirm their contributions to the paper as follows: A.M.: Conceptualization, investigation, data curation, validation, writing – original draft; Z.H.E.: Methodology, data curation, formal analysis, validation; H.A.: Methodology, formal analysis, validation, editing; Samin Sharafian: Investigation, validation, editing; Sabahat Haghi: Investigation, validation, editing; N.R.: Investigation, validation, review and editing; R.Y.: Supervision, investigation, validation, writing – review and editing; M.T.: Supervision, investigation, validation, writing – review and editing. All authors read and approved the final manuscript.

## Figures and Tables

**Fig. (1):**
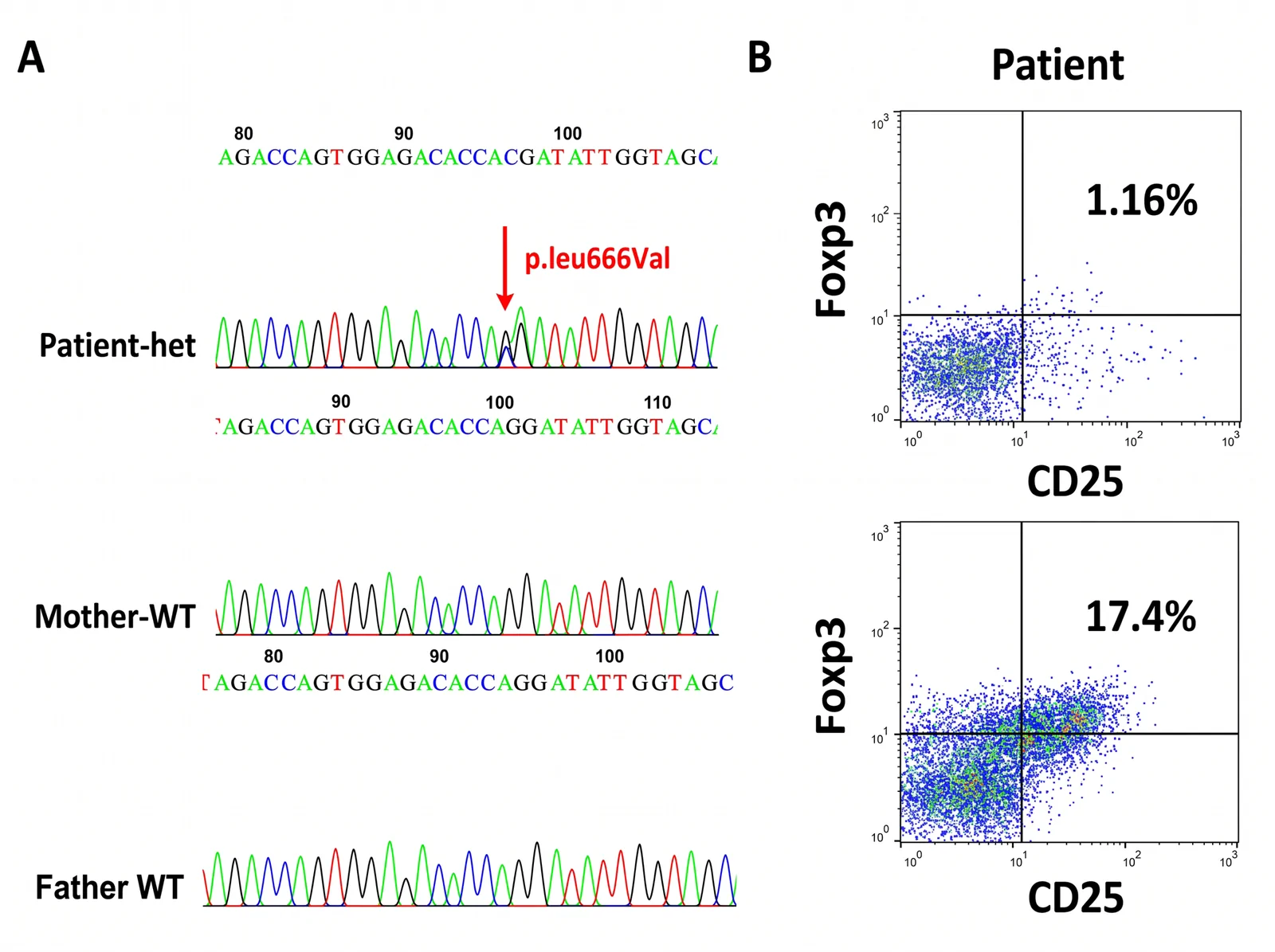
(**A**) Sanger sequencing validation of the heterozygous *de novo* STAT3 mutation in the patient and his parents. (**B**) Flow cytometric analysis of regulatory T cells (Tregs). Lymphocytes were first gated based on forward- and side-scatter characteristics. After gating on CD4^+^ CD127low/− cells, Tregs were identified as CD25^+^ FOXP3^+^. Representative plots are shown for a healthy control (left) and the patient (right), illustrating a marked reduction in Treg frequency in the patient.

**Fig. (2):**
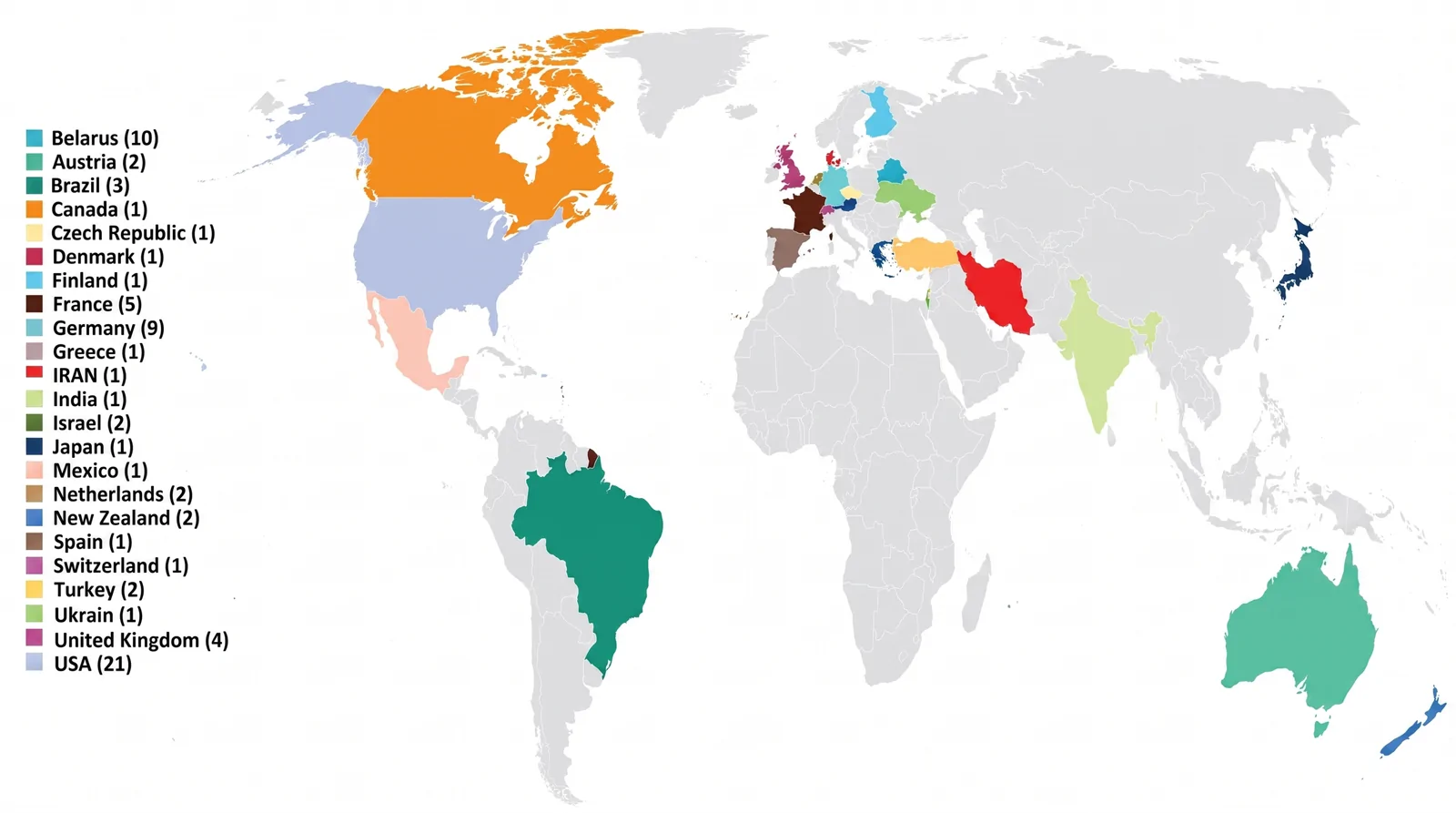
The geographical distribution of 66 reported ALPS-like patients with STAT3 GOF mutations. Each country is shown in a different color on the map, and the number of patients in each country is indicated in parentheses in the legend (Created with https://www.datawrapper.de).

**Table 1:** Laboratory and immunologic data of a patient with STAT3 GOF mutation.

**Laboratory Test**	**Values**	**Reference Values**
** Complete Blood Count **		
WBC/mm3	2350↓	4800-10800
Neutrophils (%)	6 (140)↓	25-76
Platelets (10^3/UL)	89↓	130-400
Hemoglobin (g/dL)	13.7	14-18
Lymphocytes (%)	59	21-63
Monocytes (%)	31.9↑	0-12
Eosinophil (%)	0.06	0-5
CD3+ (%)	65.2	35-78
CD4+ (%)	22.8	22-62
CD8+ (%)	38	12-36
CD4+/CD8+ ratio	0.6	1-3
CD16+ (%)	5.5	5-19
CD56+ (%)	10	3-15
CD19+ (%)	21	3-14
HLA-DR	27.7	3-15
CD45+CD3+(%)	66	60-76
CD3+CD4-CD8-DNT TCR αβ+ (%)	5.28↑	˂2% CD3+ T cells
CD3+CD4-CD8-DNT TCR αβ+(cells/mcl)	90↑	˂35
T reg cells	1.1%↓	**5.6–8.0%**
** Serum Immunoglobulins **		
IgM (mg/dL)	436↑	42-255
IgG (mg/dL)	19300↑	462-1682
IgA (mg/dL)	314↑	34-274
IgE (IU/ml)	258↑	˂115
** Infections Panel **		
Anti EBV CA IgM	Neg	Neg
Anti EBV CA IgG	POS (2.15)	Neg
HIV 1&2 Ab	Neg	Neg
EBV PCR	Neg	Neg
CMV PCR	Neg	Neg
TB complex PCR	Neg	Neg
Direct Coombs	Neg	Neg
PANCA	Neg	Neg
CANCA	Neg	Neg
FANA	Neg	Neg
ESR (mm/h)	28↑	0-10
CRP (mg/l)	6	˂6
** Thyroid Markers **		
TSH (miu/dl)	4.2	1.7-9.1
T4(mcg/dl)	13.2	6.8-14.5
T3(ng/dl)	222	94-240
**Liver function**		
AST	17	10-40
ALT	9	10-40
ALK	365	180-1200
Vit B12(pg/ml)	1020↑	193-982
** Lymphocyte Transformation Test (LTT) **		
BCG	1.4↓	≥2.5
DT	1↓	≥2.5
*Candida*	1↓	≥2.5

**Abbreviations:** *WBC (white blood cells), CD3 (cluster of differentiation), Treg (T regulatory cells), Ig (Immunoglobulin), EBV (Epstein-Barr virus), HIV (Human immunodeficiency virus), PCR (Polymerase chain reaction), TB (Tuberculosis), PANCA (Perinuclear anti-neutrophil cytoplasmic antibodies), CANCA (cytoplasmic antineutrophil cytoplasmic antibodies, FANA (fluorescent antinuclear antibody), ESR (erythrocyte sedimentation rate), CRP (C-reactive protein), TSH (Thyroid-stimulating hormone), AST (aspartate aminotransferase), ALT (alanine aminotransferase), ALK (alkaline phosphatase), BCG (Bacillus Calmette-Guerin), DT (diphtheria, tetanus), GOF (gain-of-function).

**Table 2:** Demographic Data in ALPS-like Patients with STAT3 GOF mutations.

**No.**	**Age (Year)**	**AOO** **(Year)**	**AOD** **(Year)**	**Sex**	**Dead/Alive**
1	18	5	-	F	Alive
2	28	4	27	F	Alive
3	33	10	33	F	Alive
4	41	-	-	M	Alive
5	20	-	-	M	Alive
6	12	1	9	M	Alive
7	18	9	16	M	Alive
8	6.5	4.5	5.5	F	Alive
9	12	2	9	F	Alive
10	29	1	28	F	Alive
11	13	4	9	F	Alive
12	23	1	20	-	Alive
13	12	0	11	M	Alive
14	47	0.25	45	M	Alive
15	15	2	11	F	Dead
16	9	5	6	M	Alive
17	24	10	22	M	Alive
18	55	-	11	F	Alive
19	22	NA	20	M	Dead
20	17	2	14	M	Alive
21	17	11	17	F	Alive
22	-	3	-	F	Alive
23	12	2	11	M	Alive
24	28	4	27	M	Alive
25	14	10	12	F	Alive
26	18	-	3	M	Alive
27	16	1	14	F	Alive
28	32	13	32	M	Alive
29	48	17	43	F	Alive
30	25	11	20	F	Dead
31	22	-	22	F	Alive
32	11	4	10	M	Alive
33	19	0	15	F	Alive
34	-	2	-	F	Alive
35	12	3	10	M	Alive
36	35	1	35	M	Alive
37	18	3	18	M	Alive
38	21	2	17	-	Alive
39	3.5	1.75	3	M	Alive
40	44	6	44	M	Alive
41	7	1	5	M	Alive
42	19	1	17	F	Dead
43	31	7	31	M	Alive
44	26	1	26	F	Alive
45	24	12	24	F	Alive
46	16	7	15	M	Dead
47	41	23	35	F	Alive
48	23	0.4	20	F	Alive
49	22	-	-	M	Alive
50	18	2	14	F	-
51	6	5	5	F	Alive
52	43	26	42	M	Alive
53	7	0.2	6.5	-	Alive
54	15	12	15	M	Alive
55	15	-	-	F	Alive
56	20.5	3.6	-	M	Dead
57	14	1	11	M	Alive
58	11	3	8	F	Alive
59	-	2	3	M	Alive
60	11	5	8	F	Alive
61	22	8	15	M	Alive
62	21	0.75	20	M	Alive
63	18	0.25	16	M	Alive
64	40	18	40	F	Dead
65	7.75	6	-	M	Alive
66	16	1	11	NR	Alive
Median (IQR), years	18 (13-26)	3 (1-7)	15 (10-25)		
Percentage				F (49.1), M (50.9)	

**Abbreviation:** F: Female, M: Male, NA: Not reported, AOO: Age Of Onset, AOD: Age Of Diagnosis.

**Table 3:** Clinical presentation in ALPS-like patients with STAT3 GOF mutations.

**P. No.**	**Lymphade** **nopathy**	**Spleno** **megaly**	**Hepatomegaly**	**Types of Autoimmune Diseases**	**Skin Disease**	**FTT**	**Viral** **Iinfection**	**Bacterial Infection**	**Refs.**
1	Yes	Yes	-	ITP	Yes	Yes	Yes	Yes	[23]
2	Yes	Yes	-	ITP	Yes	Yes	Yes	Yes	[4]
3	Yes	Yes	-	ITP	Yes	Yes	Yes	Yes	[18]
4	Yes	Yes	-	ITP	No	No	Yes	No	[4]
5	Yes	Yes	-	ITP/AIN	No	No	No	No	[18]
6	Yes	Yes	-	ITP/ AIHA	Yes	No	Yes	No	[4]
7	Yes	Yes	-	ITP/ AIHA/AIN	No	No	Yes	Yes	[4]
8	Yes	Yes	-	AIHA	Yes	Yes	Yes	No	[20]
9	Yes	Yes	-	AIHA	No	Yes	No	No	[4]
10	Yes	Yes	-	ITP	No	No	No	No	[4]
11	Yes	Yes	-	AIHA	Yes	No	No	Yes	[4]
12	Yes	Yes	-	ITP/AIHA	Yes	Yes	Yes	Yes	[4]
13	Yes	Yes	-	AIN	No	Yes	Yes	Yes	[4]
14	Yes	Yes	-	ITP/ AIHA	No	Yes	Yes	Yes	[4]
15	Yes	Yes	-	ITP/ AIHA/AIN	No	Yes	Yes	No	[7]
16	Yes	Yes	-	ITP	No	No	No	No	[18]
17	Yes	Yes	-	ITP	Yes	No	No	Yes	[4]
18	Yes	Yes	-	ITP/ AIHA	Yes	No	Yes	Yes	[23]
19	Yes	Yes	-	ITP/AIN	Yes	yes	No	Yes	[4]
20	Yes	Yes	-	ITP/ AIHA	No	No	No	Yes	[4]
21	Yes	Yes	-	ITP/ AIHA/AIN	Yes	Yes	No	No	[4]
22	Yes	Yes	-	ITP/ AIHA	Yes	Yes	Yes	No	[5]
23	Yes	Yes	-	ITP/ AIHA/AIN	No	No	No	Yes	[4]
24	Yes	Yes	-	ITP/ AIHA/AIN	Yes	No	Yes	Yes	[4]
25	Yes	Yes	-	AIHA/AIN	No	Yes	No	Yes	[18]
26	Yes	Yes	-	ITP/ AIHA	Yes	Yes	Yes	Yes	[23]
27	Yes	Yes	-	ITP/AIN	Yes	Yes	Yes	Yes	[4]
28	Yes	Yes	No	ITP/ AIHA/ AIN	Yes	No	Yes	Yes	[19]
29	Yes	Yes	-	ITP	Yes	Yes	Yes	Yes	[4]
30	Yes	Yes	-	ITP	Yes	No	Yes	Yes	[4]
31	Yes	Yes	-	AIHA/AIN	No	No	Yes	Yes	[4]
32	Yes	Yes	-	ITP/ AIHA	No	No	Yes	No	[4]
33	Yes	Yes	Yes	AIHA	No	Yes	Yes	Yes	[29]
34	Yes	Yes	-	ITP/ AIHA/AIN	No	No	No	No	[5]
35	Yes	Yes	-	ITP/AIN	Yes	Yes	No	No	[18]
36	Yes	Yes	-	ITP	Yes	No	No	Yes	[4]
37	Yes	Yes	-	ITP/ AIHA/AIN	No	Yes	No	No	[20]
38	Yes	Yes	-	ITP/ AIHA	No	Yes	No	No	[21]
39	Yes	Yes	-	ITP/ AIHA/AIN	Yes	Yes	No	Yes	[4]
40	Yes	Yes	-	AIHA	No	No	No	Yes	[4]
41	Yes	Yes	-	AIHA	No	No	Yes	Yes	[18]
42	Yes	Yes	Yes	AIHA	Yes	Yes	Yes	Yes	[29]
43	Yes	Yes	Yes	ITP/ AIHA	No	No	Yes	Yes	[19]
44	Yes	Yes	Yes	ITP/ AIHA/AIN	Yes	Yes	No	No	[19]
45	Yes	Yes	Yes	ITP/AIN	No	No	No	No	[19]
46	Yes	Yes	-	ITP	Yes	Yes	Yes	Yes	[7]
47	Yes	Yes	-	AIHA	No	No	No	No	[4]
48	Yes	Yes	-	ITP/ AIHA/AIN	No	Yes	Yes	Yes	[20]
49	Yes	Yes	-	ITP/ AIHA	Yes	Yes	No	Yes	[4]
50	Yes	Yes	-	ITP/ AIHA	No	Yes	Yes	Yes	[21]
51	Yes	Yes	-	ITP/ AIHA/AIN	No	Yes	No	No	[4]
52	Yes	Yes	-	ITP/ AIHA/AIN	No	Yes	Yes	Yes	[18]
53	Yes	Yes	-	ITP/ AIHA	No	Yes	Yes	Yes	[4]
54	Yes	Yes	Yes	ITP/ AIHA/AIN	Yes	No	Yes	Yes	[22]
55	Yes	Yes	-	ITP/ AIHA/AIN	No	No	No	Yes	[4]
56	Yes	Yes	-	ITP/ AIHA/AIN	No	No	Yes	Yes	[21]
57	Yes	Yes	-	ITP/AIN	No	Yes	No	No	[18]
58	Yes	Yes	-	ITP/ AIHA/AIN	No	Yes	Yes	Yes	[4]
59	Yes	Yes	Yes	ITP/AIN	Yes	No	No	No	[24]
60	Yes	Yes	-	ITP/AIN/AIHA	No	Yes	No	No	[18] [20]
61	Yes	Yes	-	ITP/AIN	No	Yes	No	No	[4]
62	Yes	Yes	-	ITP	No	No	No	No	[4]
63	Yes	Yes	-	ITP/ AIHA	Yes	No	Yes	Yes	[18]
64	Yes	Yes	-	AIHA	Yes	No	Yes	Yes	[4]
65	Yes	Yes	-	ITP/ AIHA/AIN	No	Yes	No	No	[4]
66	Yes	Yes	-	ITP	No	No	No	No	[4]
Percentage	100	100	Yes (87.5), No (12.5)	ITP (81.85), AIN (45.4), AIHA (66.6)	42	53	53	59.1	

**Abbreviations:** FTT; Failure to Thrive, ITP; Immune Thrombocytopenic Purpura, AIHA; Autoimmune Hemolytic Anemia, AIN; Autoimmune Neutropenia, Ref; Reference.

**Table 4:** Laboratory and genetic findings in ALPS-like patients with STAT3 GOF mutations.

**N**	**CD4 (/μl)**	**CD8 (/μl)**	**Treg Cells**	**DNT**	**IgG (mg/ml)**	**IgA (mg/ml)**	**IgM (mg/ml)**	**Protein Change**	**Type of Mutation**	**Zygosity**	**Refs.**
1	-	-	↓	↑	340↓	8↓	20↓	Arg103Trp	missense	hetero	[23]
2	84	78	-	-	559↓	99	138	Arg103Trp	missense	hetero	[4]
3	154	29	-	↑	300↓	30↓	20↓	Arg103Gln	missense	hetero	[18]
4	430	228	-	↑	110↑	8↓	5↓	Arg107Trp	missense	hetero	[4]
5	-	-	↓	↑	-	-	-	Arg107Gln	missense	hetero	[18]
6	328	348	-	↑	125↓	53	33↓	Arg152Trp	missense	hetero	[4]
7	425	925	N	↑	420↓	92	100	Arg152Trp	missense	hetero	[4]
8	24	-	↑	↑	1350	200	189	Arg152Trp	missense	hetero	[20]
9	1087	710	N	↑	1564	90	95	Arg152Trp	missense	hetero	[4]
10	508	389	N	↑	605↓	118	57.6	Arg152Trp	missense	hetero	[4]
11	659	573	N	↑	1110	101	75	Arg152Trp	missense	hetero	[4]
12	456	339	N	↑	707	210	47	Arg152Trp	missense	hetero	[4]
13	592	2328	↓	↑	243↓	11↓	7↓	Glu166Asp	missense	hetero	[4]
14	556	878	-	-	382↓	10↓	125	Glu166Lys	missense	hetero	[4]
15	206	667	↓	↑	497↓	107	71	Phe174Ser	Missense	hetero	[7]
16	-	-	↓	↑	-	-	-	Met206Arg	missense	hetero	[18]
17	624	498	-	-	228↓	˂1↓	˂17↓	Val218Ala	missense	hetero	[4]
18	1254	902	-	-	60↓	7↓	20↓	Arg246Gln	missense	hetero	[23]
19	557	311	-	-	355↓	˂7↓	˂7↓	Arg278Cys	missense	hetero	[4]
20	552	445	N	↑	1670↑	565↑	58	Arg278Cys	missense	hetero	[4]
21	360	555	-	↑	1060	110	158	Arg278His	missense	hetero	[4]
22	-	-	↓	↑	150↓	22.8↓	1460↑	Arg278His	missense	hetero	[5]
23	597	718	N	↑	464↓	˂6.3↓	˂17.4↓	Arg278His	missense	hetero	[4]
24	-	-	↓	↑	450↓	114	81	Arg278His	missense	hetero	[4]
25	424	319	-	-	316↓	24↓	17↓	Leu287Val	missense	hetero	[18]
26	337	276	-	-	681↓	˂7↓	37	Phe313Leu	missense	hetero	[23]
27	192	294	-	N	348↓	219	18.9↓	Gln344His	missense	hetero	[4]
28	-	-	-	↑	248↓	9↓	˂5↓	Val353Phe	missense	hetero	[19]
29	840	2380	-	-	520↓	50↓	60	Thr360Cys	missense	hetero	[4]
30	260	560	-	-	78↓	17↓	93	Thr360Cys	missense	hetero	[4]
31	133	407	-	↑	699	0↓	28↓	Gln361Arg	missense	hetero	[4]
32	141	1569	-	↑	845	98	60	Thr389Arg	missense	hetero	[4]
33	666	1790	↓	N	280↓	21↓	144	Lys392Arg	missense	hetero	[29]
34	-	-	-	N	572	27↓	31	Met394Thr	missense	hetero	[5]
35	-	-	-	↑	-	-	-	Asn401Asp	missense	hetero	[18]
36	-	-	-	-	110↓	26↓	88	Phe408Leu	missense	hetero	[4]
37	160	370	-	-	1390	33↓	354	Gly421Arg	missense	hetero	[20]
38	496	416	-	↑	440↓	55↓	90	Gly421Arg	missense	hetero	[21]
39	1325	1108	-	-	1651↑	288↑	405↑	Gly422Arg	missense	hetero	[4]
40	339	165	-	↑	82↓	288	25↓	Glu506Lys	missense	hetero	[4]
41	1048	1936	-	↑	1509	163	70	Gln635Leu	missense	hetero	[18]
42	789	570	↓	-	580↓	167	338	Lys658Asn	missense	hetero	[29]
43	-	-	-	N	↓	↓	↓	Thr663Ile	missense	hetero	[19]
44	-	-	-	N	Normal	-	-	Ala703Thr	missense	hetero	[19]
45	-	-	-	-	Normal	-	-	Ala703Thr	missense	hetero	[19]
46	198	231	↓	↑	476↓	˂8↓	20↓	Pro715Leu	missense	hetero	[7]
47	210	335	-	-	5640↑	2310↑	1190↑	Pro715Leu	missense	hetero	[4]
48	480	716	↓	N	587↓	39↓	115	Pro715Leu	missense	hetero	[20]
49	315	252	N	N	437↓	71	22↓	Pro715Leu	missense	hetero	[4]
50	250	165	-	N	Normal	-	-	Pro715Leu	missense	hetero	[21]
51	156	205	-	↑	1820↑	120	155	Pro715Leu	missense	hetero	[4]
52	291	213	-	-	865	75	65	Thr716Met	missense	hetero	[18]
53	1867	604	-	↑	731	213↑	79.9	Thr716Met	missense	hetero	[4]
54	647	317	-	↑	400↓	115	93	Thr716Met	missense	hetero	[22]
55	530	460	-	↑	484↓	2↓	83	Thr716Met	missense	hetero	[4]
56	648	720	-	↑	900	-	-	Thr716Met	missense	hetero	[21]
57	-	-	↓	↑	808	-	-	Thr716Met	missense	hetero	[18]
58	-	-	-	-	-	--	-	Thr716Met	missense	hetero	[4]
59	-	-	↓	N	1562	42.7	155	Thr716Met	missense	hetero	[24]
60	-	-	-	↑	-	28↓	34↓	Thr716Met	missense	hetero	[18] [20]
61	300	680	-	↑	470↓	70	190	Thr716Met	missense	hetero	[4]
62	487	823	↑	↑	563↓	169	230	Thr716Met	missense	hetero	[4]
63	373	265	↓	↑	390↓	63	64	Thr716Met	missense	hetero	[18]
64	880	1620	-	-	˂300↓	˂100	240	Thr716Met	missense	hetero	[4]
65	446	685	-	↑	2010↑	378↑	167	Thr716Met	missense	hetero	[4]
66	768	888	-	↑	505	60	30↓	Thr716Met	missense	hetero	[4]
Median (IQR), years	451 (257.5-647.3)	498 (302.5-771)	↓ (58) ↑ (8) N (40)	↓ (60.4) N (3.6)	501(342-860)	70.5(25.5-130.8)	77.4(31.5-152.3)				
Percentage								Thr716Met (22.7)	100	100	

**Abbreviations:** ↑; increased, ↓; decreased, N; normal, Treg (T Regulatory), DNT (Double Negative T Cell), hetro (heterozygous), Alanine (Ala), C; cysteine (Cys), D; aspartic acid (Asp), E; glutamic acid (Glu), F; phenylalanine (Phe), G, glycine (Gly), H; histidine (His), I; isoleucine (Ile), K; lysine (Lys), L; leucine (Leu), M; methionine (Met), N; asparagine (Asn), P, proline (Pro); Q; glutamine (Gln), R; arginine (Arg), S; serine (Ser), T; threonine (Thr), V; valine (Val), W; tryptophan (Trp), Y; tyrosine (Tyr).

**Table 5:** The status of treatment in ALPS-like patients with STAT3 GOF mutations.

**P. No.**	**IVIG**	**JAK Inhibitor**	**HSCT**
1	Yes	Yes	No
2	Yes	No	No
3	Yes	No	No
4	Yes	No	No
5	No	No	No
6	Yes	No	No
7	Yes	No	No
8	Yes	No	No
9	Yes	No	No
10	Yes	No	No
11	No	Yes	No
12	Yes	No	No
13	Yes	No	No
14	Yes	No	No
15	Yes	Yes	No
16	Yes	No	No
17	Yes	No	No
18	Yes	No	No
19	No	No	Yes
20	Yes	No	No
21	Yes	Yes	Yes
22	No	No	No
23	Yes	No	No
24	Yes	No	No
25	Yes	No	No
26	Yes	No	No
27	Yes	No	No
28	Yes	No	No
29	Yes	Yes	No
30	Yes	Yes	No
31	Yes	Yes	No
32	Yes	Yes	No
33	Yes	No	Yes
34	Yes	No	No
35	Yes	No	No
36	Yes	No	No
37	No	No	No
38	Yes	No	No
39	Yes	Yes	No
40	Yes	No	No
41	No	No	Yes
42	Yes	No	Yes
43	Yes	No	No
44	No	No	No
45	No	No	No
46	Yes	Yes	No
47	No	No	No
48	Yes	No	No
49	Yes	Yes	No
50	Yes	No	No
51	Yes	Yes	No
52	Yes	No	No
53	Yes	No	No
54	Yes	No	No
55	Yes	No	No
56	Yes	No	No
57	No	No	No
58	Yes	No	No
59	No	No	No
60	Yes	No	No
61	Yes	No	No
62	Yes	Yes	No
63	Yes	No	No
64	Yes	No	No
65	Yes	Yes	No
66	Yes	No	No
percentage	Yes (83.3), No (16.7)	Yes (21.2), No (78.8)	Yes (7.5), No (92.5)

**Abbreviations:** *IVIG (Intravenous Immunoglobulin), HSCT (Hematopoietic stem cell transplantation), JAK)Janus kinases).

## Data Availability

The data supporting our findings are available from the corresponding author upon reasonable request.

## LIST OF ABBREVIATIONS

ALPS: Autoimmune Lymphoproliferative Syndrome
FASL: FAS Ligand
CASP8: Caspase 8
CASP10: Caspase 10
STAT3: Signal Transducer and Activator of Transcription 3
IFNs: Interferons
GOF: Gain-of-Function
JAKs: Janus Kinases
IEI: Inborn Errors of Immunity
LTT: Lymphocyte Transformation Test
ACMG: American College of Medical Genetics and Genomics
PBMCs: Peripheral Blood Mononuclear Cells
AIHA: Autoimmune Hemolytic Anemia
ITP: Immune Thrombocytopenic Purpura
AN: Autoimmune Neutropenia
IVIG: Intravenous Immunoglobulin
BCG: Bacillus Calmette–Guérin
DT: Diphtheria Toxoid
Treg: Regulatory T Cell
FTT: Failure to Thrive
CSR: Class Switch Recombination
HSCT: Hematopoietic Stem Cell Transplantation
ESID: European Society for Immunodeficiencies
T-LGLL: T-Cell Large Granular Lymphocytic Leukemia
mTOR: Mammalian Target of Rapamycin

## References

[1] Palmisani E., Miano M., Grossi A., Lanciotti M., Lupia M., Terranova P., Ceccherini I., Montanari E., Calvillo M., Pierri F., Micalizzi C., Maggiore R., Guardo D., Zanardi S., Facchini E., Maggio A., Mastrodicasa E., Corti P., Russo G., Pillon M., Farruggia P., Cesaro S., Barone A., Tosetti F., Ramenghi U., Crescenzio N., Bleesing J., Dufour C., Fioredda F. (2023). Autoimmune lymphoproliferative syndrome (ALPS) disease and ALPS phenotype: Are they two distinct entities?. HemaSphere.

[2] López-Nevado M., González-Granado L.I., Ruiz-García R., Pleguezuelo D., Cabrera-Marante O., Salmón N., Blanco-Lobo P., Domínguez-Pinilla N., Rodríguez-Pena R., Sebastián E., Cruz-Rojo J., Olbrich P., Ruiz-Contreras J., Paz-Artal E., Neth O., Allende L.M. (2021). Primary immune regulatory disorders with an autoimmune lymphoproliferative syndrome-like phenotype: Immunologic evaluation, early diagnosis and management.. Front. Immunol..

[3] Hafezi N., Zaki-Dizaji M., Nirouei M., Asadi G., Sharifinejad N., Jamee M., Erfan Rasouli S., Hamedifar H., Sabzevari A., Chavoshzadeh Z., Yazdani R., Abolhassani H., Aghamohammadi A., Azizi G. (2021). Clinical, immunological, and genetic features in 780 patients with autoimmune lymphoproliferative syndrome (ALPS) and ALPS‐like diseases: A systematic review.. Pediatr. Allergy Immunol..

[4] Leiding J.W., Vogel T.P., Santarlas V.G.J., Mhaskar R., Smith M.R., Carisey A., Vargas-Hernández A., Silva-Carmona M., Heeg M., Rensing-Ehl A., Neven B., Hadjadj J., Hambleton S., Ronan Leahy T., Meesilpavikai K., Cunningham-Rundles C., Dutmer C.M., Sharapova S.O., Taskinen M., Chua I., Hague R., Klemann C., Kostyuchenko L., Morio T., Thatayatikom A., Ozen A., Scherbina A., Bauer C.S., Flanagan S.E., Gambineri E., Giovannini-Chami L., Heimall J., Sullivan K.E., Allenspach E., Romberg N., Deane S.G., Prince B.T., Rose M.J., Bohnsack J., Mousallem T., Jesudas R., Santos Vilela M.M.D., O’Sullivan M., Pachlopnik Schmid J., Průhová Š., Klocperk A., Rees M., Su H., Bahna S., Baris S., Bartnikas L.M., Chang Berger A., Briggs T.A., Brothers S., Bundy V., Chan A.Y., Chandrakasan S., Christiansen M., Cole T., Cook M.C., Desai M.M., Fischer U., Fulcher D.A., Gallo S., Gauthier A., Gennery A.R., Gonçalo Marques J., Gottrand F., Grimbacher B., Grunebaum E., Haapaniemi E., Hämäläinen S., Heiskanen K., Heiskanen-Kosma T., Hoffman H.M., Gonzalez-Granado L.I., Guerrerio A.L., Kainulainen L., Kumar A., Lawrence M.G., Levin C., Martelius T., Neth O., Olbrich P., Palma A., Patel N.C., Pozos T., Preece K., Lugo Reyes S.O., Russell M.A., Schejter Y., Seroogy C., Sinclair J., Skevofilax E., Suan D., Suez D., Szabolcs P., Velasco H., Warnatz K., Walkovich K., Worth A., Aleshkevich S., Allende L.M., Atkinson T.P., Atschekzei F., Aydemir S., Aygunes U., Barlogis V., Baumann U., Belko J., Bezrodnik L., Biebl A., Broderick L., Bunin N.J., Caldirola M.S., Castelle M., Celmeli F., Charbonnier L-M., Chatila T.A., Chellapandian D., Cokugras H., Conlon N., Cox F., Crickx E., Dalgic B., ASH Dalm V., Danielian S., Dominguez-Pinilla N., Dujovny T., Ebbo M., Eken A., Esty B., Fabre A., Fischer A., Hannibal M., Huppert L., Ikeda M.D., Jolles S., Jolly K.W., Jones N., Kanariou M., Karakoc-Aydiner E., Karamantziani T., Kelaidi C., Keogan M., Pac Kisaarslan A., Kiykim A., Klocperk A., Kotsonis K., Kuzmenko N., Leroy S., Lianou D., Longhurst H., Lorenz M.R., Maffucci P., Manson A., Marchal S., Malphettes M., Marega L.F., Mauracher A.A., Meesilpavikai K., Miller H., Mombourquette J., Morgan N.G., Mukhina A., Nathalie A., Nelken B., Nolan D., Norlin A-C., Oleastro M., Ozcan A., Pasquet M., Pegler J.R., Picard C., Polychronopoulou S., Quartier P., Quesada J.F., Ramakers J., Randall K.L., Rao V.K., Remiker A., Resin G., Richmond P., Rieux-Laucat F., Rodina Y., Rohrlich P., Sachs J., Sakovich I., Santarlas C., Sari S., Sawicki G., Schauer U., Scheffler Mendoza S.C., Schvetz O., Schmidt R.E., Schwarz K., Sediva A., Sinclair K., Slatter M., Sleasman J., Stergiou K., Suratannon N., Tanita K., Thompson G., Travis S., Trojan T., Tsinti M., Unal E., Urdinez L., Vazquez-Gomez F., Villa M., Weinrich M., Weiss M.J., Wright B., Yilmaz E., Zachova R., Zhang Y., Seppänen M.R.J., Torgerson T.R., Sogkas G., Ehl S., Tangye S.G., Cooper M.A., Milner J.D., Forbes Satter L.R. (2023). Monogenic early-onset lymphoproliferation and autoimmunity: Natural history of STAT3 gain-of-function syndrome.. J. Allergy Clin. Immunol..

[5] Nabhani S., Schipp C., Miskin H., Levin C., Postovsky S., Dujovny T., Koren A., Harlev D., Bis A.M., Auer F., Keller B., Warnatz K., Gombert M., Ginzel S., Borkhardt A., Stepensky P., Fischer U. (2017). STAT3 gain-of-function mutations associated with autoimmune lymphoproliferative syndrome like disease deregulate lymphocyte apoptosis and can be targeted by BH3 mimetic compounds.. Clin. Immunol..

[6] Schmitt E.G., Toth K.A., Risma S.I., Kolicheski A., Saucier N., Berríos R.J.F., Greenberg Z.J., Leiding J.W., Bleesing J.J., Thatayatikom A., Schuettpelz L.G., Edwards J.R., Vogel T.P., Cooper M.A. (2022). A human STAT3 gain-of-function variant confers T cell dysregulation without predominant Treg dysfunction in mice.. JCI Insight.

[7] Forbes L. R., Vogel T. P., Cooper M. A., Castro-Wagner J., Schussler E., Weinacht K. G., Plant A. S., Su H. C., Allenspach E. J., Slatter M., Abinun M., Lilic D., Cunningham-Rundles C., Eckstein O., Olbrich P., Guillerman R. P., Patel N. C., Demirdag Y. Y., Zerbe C., Freeman A. F., Holland S. M., Szabolcs P., Gennery A., Torgerson T. R., Milner J. D., Leiding J. W. (2018). Jakinibs for the treatment of immune dysregulation in patients with gain-of-function signal transducer and activator of transcription 1 (STAT1) or STAT3 mutations.. J. Allergy Clin. Immunol..

[8] Matson D.R., Yang D.T. (2020). Autoimmune Lymphoproliferative Syndrome: An overview.. Arch. Pathol. Lab. Med..

[9] Oliveira J.B., Bleesing J.J., Dianzani U., Fleisher T.A., Jaffe E.S., Lenardo M.J., Rieux-Laucat F., Siegel R.M., Su H.C., Teachey D.T., Rao V.K. (2010). Revised diagnostic criteria and classification for the autoimmune lymphoproliferative syndrome (ALPS): Report from the 2009 NIH International Workshop.. Blood.

[10] Magerus A., Rensing-Ehl A., Rao V.K., Teachey D.T., Rieux-Laucat F., Ehl S. (2024). Autoimmune lymphoproliferative immunodeficiencies (ALPIDs): A proposed approach to redefining ALPS and other lymphoproliferative immune disorders.. J. Allergy Clin. Immunol..

[11] Abolhassani H., Kiaee F., Tavakol M., Chavoshzadeh Z., Mahdaviani S.A., Momen T., Yazdani R., Azizi G., Habibi S., Gharagozlou M., Movahedi M., Hamidieh A.A., Behniafard N., Nabavi M., Bemanian M.H., Arshi S., Molatefi R., Sherkat R., Shirkani A., Amin R., Aleyasin S., Faridhosseini R., Jabbari-Azad F., Mohammadzadeh I., Ghaffari J., Shafiei A., Kalantari A., Mansouri M., Mesdaghi M., Babaie D., Ahanchian H., Khoshkhui M., Soheili H., Eslamian M.H., Cheraghi T., Dabbaghzadeh A., Tavassoli M., Kalmarzi R.N., Mortazavi S.H., Kashef S., Esmaeilzadeh H., Tafaroji J., Khalili A., Zandieh F., Sadeghi-Shabestari M., Darougar S., Behmanesh F., Akbari H., Zandkarimi M., Abolnezhadian F., Fayezi A., Moghtaderi M., Ahmadiafshar A., Shakerian B., Sajedi V., Taghvaei B., Safari M., Heidarzadeh M., Ghalebaghi B., Fathi S.M., Darabi B., Bazregari S., Bazargan N., Fallahpour M., Khayatzadeh A., Javahertrash N., Bashardoust B., Zamani M., Mohsenzadeh A., Ebrahimi S., Sharafian S., Vosughimotlagh A., Tafakoridelbari M., Rahimi M., Ashournia P., Razaghian A., Rezaei A., Mamishi S., Parvaneh N., Rezaei N., Hammarström L., Aghamohammadi A. (2018). Fourth update on the Iranian National Registry of primary immunodeficiencies: Integration of molecular diagnosis.. J. Clin. Immunol..

[12] Abolhassani H., Chou J., Bainter W., Platt C.D., Tavassoli M., Momen T., Tavakol M., Eslamian M.H., Gharagozlou M., Movahedi M., Ghadami M., Hamidieh A.A., Azizi G., Yazdani R., Afarideh M., Ghajar A., Havaei A., Chavoshzadeh Z., Mahdaviani S.A., Cheraghi T., Behniafard N., Amin R., Aleyasin S., Faridhosseini R., Jabbari-Azad F., Nabavi M., Bemanian M.H., Arshi S., Molatefi R., Sherkat R., Mansouri M., Mesdaghi M., Babaie D., Mohammadzadeh I., Ghaffari J., Shafiei A., Kalantari N., Ahanchian H., Khoshkhui M., Soheili H., Dabbaghzadeh A., Shirkani A., Nasiri Kalmarzi R., Mortazavi S.H., Tafaroji J., Khalili A., Mohammadi J., Negahdari B., Joghataei M.T., al-Ramadi B.K., Picard C., Parvaneh N., Rezaei N., Chatila T.A., Massaad M.J., Keles S., Hammarström L., Geha R.S., Aghamohammadi A. (2018). Clinical, immunologic, and genetic spectrum of 696 patients with combined immunodeficiency.. J. Allergy Clin. Immunol..

[13] Karimi E., Mahmoudian F., Reyes S.O.L., Bargir U.A., Madkaikar M., Artac H., Sabzevari A., Lu N., Azizi G., Abolhassani H. (2021). Approach to genetic diagnosis of inborn errors of immunity through next-generation sequencing.. Mol. Immunol..

[14] Richards S., Aziz N., Bale S., Bick D., Das S., Gastier-Foster J., Grody W.W., Hegde M., Lyon E., Spector E., Voelkerding K., Rehm H.L. (2015). Standards and guidelines for the interpretation of sequence variants: A joint consensus recommendation of the American College of Medical Genetics and Genomics and the Association for Molecular Pathology.. Genet. Med..

[15] Bousfiha A.A., Jeddane L., Moundir A., Poli M.C., Aksentijevich I., Cunningham-Rundles C., Hambleton S., Klein C., Morio T., Picard C., Puel A., Rezaei N., Seppänen M.R.J., Somech R., Su H.C., Sullivan K.E., Torgerson T.R., Tangye S.G., Meyts I. (2025). The 2024 update of IUIS phenotypic classification of human inborn errors of immunity.. Journal of Human Immunity.

[16] Vogel T.P., Leiding J.W., Cooper M.A., Forbes Satter L.R. (2023). STAT3 gain-of-function syndrome.. Front Pediatr..

[17] Cortesi M., Soresina A., Dotta L., Gorio C., Cattalini M., Lougaris V., Porta F., Badolato R. (2022). Pathogenesis of autoimmune cytopenias in inborn errors of immunity revealing novel therapeutic targets.. Front. Immunol..

[18] Jägle S., Heeg M., Grün S., Rensing-Ehl A., Maccari M.E., Klemann C., Jones N., Lehmberg K., Bettoni C., Warnatz K., Grimbacher B., Biebl A., Schauer U., Hague R., Neth O., Mauracher A., Pachlopnik Schmid J., Fabre A., Kostyuchenko L., Führer M., Lorenz M.R., Schwarz K., Rohr J., Ehl S. (2020). Distinct molecular response patterns of activating STAT3 mutations associate with penetrance of lymphoproliferation and autoimmunity.. Clin. Immunol..

[19] Milner J.D., Vogel T.P., Forbes L., Ma C.A., Stray-Pedersen A., Niemela J.E., Lyons J.J., Engelhardt K.R., Zhang Y., Topcagic N., Roberson E.D.O., Matthews H., Verbsky J.W., Dasu T., Vargas-Hernandez A., Varghese N., McClain K.L., Karam L.B., Nahmod K., Makedonas G., Mace E.M., Sorte H.S., Perminow G., Rao V.K., O’Connell M.P., Price S., Su H.C., Butrick M., McElwee J., Hughes J.D., Willet J., Swan D., Xu Y., Santibanez-Koref M., Slowik V., Dinwiddie D.L., Ciaccio C.E., Saunders C.J., Septer S., Kingsmore S.F., White A.J., Cant A.J., Hambleton S., Cooper M.A. (2015). Early-onset lymphoproliferation and autoimmunity caused by germline STAT3 gain-of-function mutations.. Blood.

[20] Mauracher A.A., Eekels J.J.M., Woytschak J., van Drogen A., Bosch A., Prader S., Felber M., Heeg M., Opitz L., Trück J., Schroeder S., Adank E., Klocperk A., Haralambieva E., Zimmermann D., Tantou S., Kotsonis K., Stergiou A., Kanariou M.G., Ehl S., Boyman O., Sediva A., Renella R., Schmugge M., Vavassori S., Pachlopnik Schmid J. (2020). Erythropoiesis defect observed in STAT3 GOF patients with severe anemia.. J. Allergy Clin. Immunol..

[21] Hadjadj J., Aladjidi N., Fernandes H., Leverger G., Magérus-Chatinet A., Mazerolles F., Stolzenberg M.C., Jacques S., Picard C., Rosain J., Fourrage C., Hanein S., Zarhrate M., Pasquet M., Abou Chahla W., Barlogis V., Bertrand Y., Pellier I., Colomb Bottollier E., Fouyssac F., Blouin P., Thomas C., Cheikh N., Dore E., Pondarre C., Plantaz D., Jeziorski E., Millot F., Garcelon N., Ducassou S., Perel Y., Leblanc T., Neven B., Fischer A., Rieux-Laucat F. (2019). Pediatric Evans syndrome is associated with a high frequency of potentially damaging variants in immune genes.. Blood.

[22] Suh Y.W., Horton J.C. (2019). Papilledema from gain-of-function mutations in the *STAT3* gene.. Ophthalmic Genet..

[23] Maffucci P., Filion C.A., Boisson B., Itan Y., Shang L., Casanova J.L., Cunningham-Rundles C. (2016). Genetic diagnosis using whole exome sequencing in common variable immunodeficiency.. Front. Immunol..

[24] Takagi M., Hoshino A., Yoshida K., Ueno H., Imai K., Piao J., Kanegane H., Yamashita M., Okano T., Muramatsu H., Okuno Y., Shiraishi Y., Chiba K., Tanaka H., Miyano S., Ogawa S., Hayashi Y., Kojima S., Morio T. (2018). Genetic heterogeneity of uncharacterized childhood autoimmune diseases with lymphoproliferation.. Pediatr. Blood Cancer.

[25] Faletti L., Ehl S., Heeg M. (2021). Germline STAT3 gain-of-function mutations in primary immunodeficiency: Impact on the cellular and clinical phenotype.. Biomed. J..

[26] Teramo A., Barilà G., Calabretto G., Ercolin C., Lamy T., Moignet A., Roussel M., Pastoret C., Leoncin M., Gattazzo C., Cabrelle A., Boscaro E., Teolato S., Pagnin E., Berno T., De March E., Facco M., Piazza F., Trentin L., Semenzato G., Zambello R. (2017). *STAT3* mutation impacts biological and clinical features of T-LGL leukemia.. Oncotarget.

[27] Casamayor-Polo L., López-Nevado M., Paz-Artal E., Anel A., Rieux-Laucat F., Allende L.M. (2021). Immunologic evaluation and genetic defects of apoptosis in patients with autoimmune lymphoproliferative syndrome (ALPS).. Crit. Rev. Clin. Lab. Sci..

[28] Molnár E., Radwan N., Kovács G., Andrikovics H., Henriquez F., Zarafov A., Hayman M., Linzner D., Thrasher A.J., Buckland M., Burns S.O., Gilmour K.C. (2020). Key diagnostic markers for autoimmune lymphoproliferative syndrome with molecular genetic diagnosis.. Blood.

[29] Haapaniemi E.M., Kaustio M., Rajala H.L.M., van Adrichem A.J., Kainulainen L., Glumoff V., Doffinger R., Kuusanmäki H., Heiskanen-Kosma T., Trotta L., Chiang S., Kulmala P., Eldfors S., Katainen R., Siitonen S., Karjalainen-Lindsberg M.L., Kovanen P.E., Otonkoski T., Porkka K., Heiskanen K., Hänninen A., Bryceson Y.T., Uusitalo-Seppälä R., Saarela J., Seppänen M., Mustjoki S., Kere J. (2015). Autoimmunity, hypogammaglobulinemia, lymphoproliferation, and mycobacterial disease in patients with activating mutations in STAT3.. Blood.

[30] Gu H., Shu Z., Chen Z., Deng M., Lu D., Wu R., Wang T., Mao H. (2025). Effectiveness of sirolimus for early on‐set autoimmune cytopenias of autoimmune lymphoproliferative immunodeficiencies.. Pediatr. Allergy Immunol..

[31] Sorin B., Fadlallah J., Garzaro M., Vigneron J., Bertinchamp R., Boutboul D., Oksenhendler E., Fieschi C., Malphettes M., Galicier L. (2023). Real-life use of mTOR inhibitor-based therapy in adults with autoimmune cytopenia highlights strong efficacy in relapsing/refractory multi-lineage autoimmune cytopenia.. Ann. Hematol..

