# Supplementary material for: ALPS-like Disorder Linked with STAT3 Mutation: De novo Variant with Bicytopenia and Literature Review
Source: Endocr Metab Immune Disord Drug Targets. 2026 Jun 22;26:E18715303470784. doi: 10.2174/0118715303470784260605093400 (PMC13659197; doi:10.2174/0118715303470784260605093400)
Supplement: Supplementary file 1 [file EMIDDT-26-E18715303470784_SD1.pdf]

Supplementary Material

ALPS-like Disorder Linked with STAT3 Mutation: De Novo Variant with Bicytopenia and Literature Review

Aylar Mohammadi[1], Zahra Hamidi Esfahani[1], Hassan Abolhassani[1,2], Samin Sharafian[3], Sabahat Haghi[4], Nima Rezaei1,[5], Reza Yazdani[1,5,*] and Marzieh Tavakol[6,*]

[1]Research Center for Immunodeficiencies, Pediatrics Center of Excellence, Children's Medical Center, Tehran University of Medical Sciences, Tehran, Iran; [2]Division of Clinical Immunology, Department of Biosciences and Nutrition, Karolinska Institutet, Karolinska University Hospital, Huddinge, Stockholm, Sweden; [3]Department of Allergy and Clinical Immunology, Mofid Children's Hospital, Shahid Beheshti University of Medical Sciences, Tehran, Iran; [4]Department of Pediatrics, Imam Ali Hospital, Alborz University of Medical Sciences, Karaj, Iran; [5]Primary Immunodeficiency Diseases Network (PIDNet), Universal Scientific Education and Research Network (USERN), Tehran, Iran; [6]NonCommunicable Diseases Research Center, Alborz University of Medical Sciences, Karaj, Iran

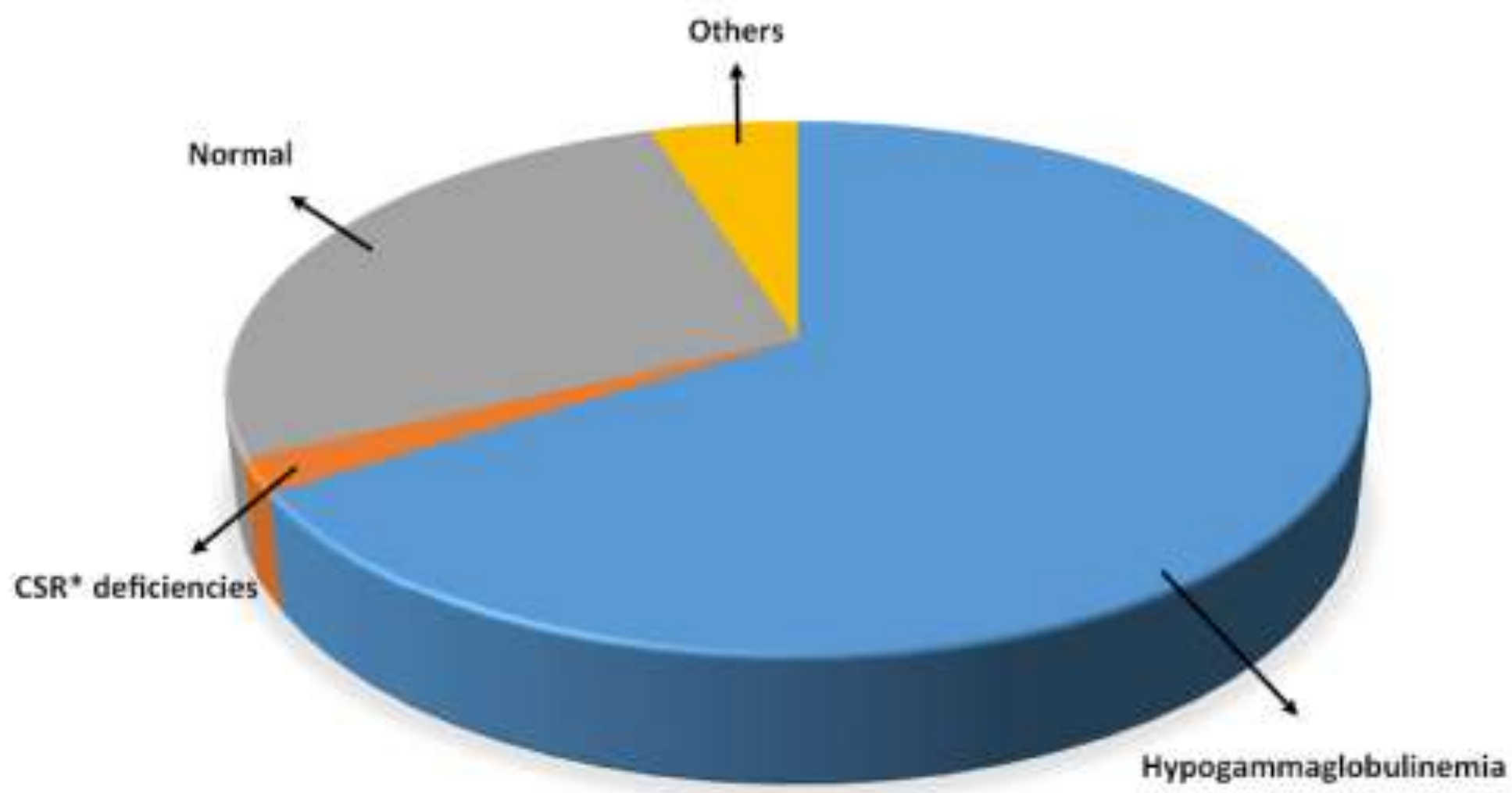

Fig. (S1).
